# Antiproliferative effect of exemestane in lung cancer cells

**DOI:** 10.1186/1476-4598-8-109

**Published:** 2009-11-24

**Authors:** Angelos Koutras, Efstathia Giannopoulou, Ismini Kritikou, Anna Antonacopoulou, TR Jeffry Evans, Athanasios G Papavassiliou, Haralabos Kalofonos

**Affiliations:** 1 Division of Oncology, Department of Medicine, University Hospital of Patras, Rion 26504, Greece; 2 Clinical Oncology Laboratory, University Hospital of Patras, Patras Medical School, Rion 26504, Greece; 3 University of Glasgow, Cancer Research UK Beatson Laboratories, Garscube Estate, Switchback Road, Glasgow G61 1BD, UK; 4 Department of Biological Chemistry, Medical School, University of Athens, 11527 Athens, Greece

## Abstract

**Background:**

Recent evidence suggests that estrogen signaling may be involved in the pathogenesis of non-small cell lung cancer (NSCLC). Aromatase is an enzyme complex that catalyses the final step in estrogen synthesis and is present in several tissues, including the lung. In the current study we investigated the activity of the aromatase inhibitor exemestane in human NSCLC cell lines H23 and A549.

**Results:**

Aromatase expression was detected in both cell lines. H23 cells showed lower protein and mRNA levels of aromatase, compared to A549 cells. Exemestane decreased cell proliferation and increased apoptosis in both cell lines, 48 h after its application, with A549 exhibiting higher sensitivity than H23 cells. Aromatase protein and mRNA levels were not affected by exemestane in A549 cells, whereas an increase in both protein and mRNA levels was observed in H23 cells, 48 h after exemestane application. Moreover, an increase in cAMP levels was found in both cell lines, 15 min after the administration of exemestane. In addition, we studied the effect of exemestane on epidermal growth factor receptor (EGFR) localization and activation. Exemestane increased EGFR activation 15 min after its application in H23 cells. Furthermore, we demonstrated a translocation of EGFR from cell membrane, 24 h after the addition of exemestane in H23 cells. No changes in EGFR activation or localization were observed in A549 cells.

**Conclusion:**

Our findings suggest an antiproliferative effect of exemestane on NSCLC cell lines. Exemestane may be more effective in cells with higher aromatase levels. Further studies are needed to assess the activity of exemestane in NSCLC.

## Background

Lung cancer is the most common cause of cancer mortality for both men and women in the United States and non-small cell lung cancer (NSCLC) accounts for 80% of all cases. The etiology of NSCLC has not yet been efficiently elucidated. Recent evidence suggests that estrogen signaling is critical for the progression of malignancies that express estrogen receptors (ER) and may also be involved in the pathogenesis of NSCLC [1-7]. In a recent review, Dubey *et al *[8] suggest that a number of principles related to the pathogenesis or even the management of the disease, may apply to both breast and lung carcinoma.

Despite previous conflicting data, recent studies have indicated that estrogen receptors alpha (ERα) and beta (ERβ) are overexpressed in certain proportions of malignant lung cells [3,5-7,9]. Moreover, stimulation of cell proliferation with estrogens has been reported in lung cancer tissue cultures, further supporting the potential biologic role of ERs in lung carcinogenesis [3-6]. A difference of ER expression between men and women has been reported in lung cancer by Fasco et al, who found higher levels of ER in female patients [10]. In addition, women with lung cancer have also higher levels of estrogens compared to healthy females of the same age [11]. It has been hypothesized that estrogens may interact with carcinogenic components of tobacco, affecting the risk for lung cancer development. A positive association between exogenous and endogenous exposure to estrogens and the development of lung adenocarcinoma has been described in women [1], even though other studies have failed to show such a correlation [12]. Furthermore, hormone replacement therapy has been associated with shorter survival in lung cancer patients [11,13]. Recent data have also provided evidence for an association between estrogen levels and outcome, and this correlation may possibly contribute to the survival advantage seen in older women with NSCLC [14].

Given the accumulating data relating to the significance of hormonal pathways in NSCLC, a role for antiestrogen treatments in the management of the disease should not be excluded. Although tamoxifen has been evaluated in combination with chemotherapy in patients with NSCLC [15,16], the agonistic activity of this agent on the ER might limit its usefulness in lung cancer [3,4,11]. Moreover, clinical studies using tamoxifen in breast cancer patients have not shown a reduction in lung cancer incidence [17]. Fulvestrant is a pure antiestrogen which has shown antiproliferative activity in estrogen-induced growth of lung cancer cells [3,5]. In view of a possible functional interaction between the ER and the epidermal growth factor receptor (EGFR) pathways in NSCLC, fulvestrant has also been evaluated in combination with EGFR tyrosine-kinase inhibitors (TKIs), both in NSCLC xenografts [4,9] and in women with NSCLC [18], with promising results. Similar studies evaluating the combination of fulvestrant with other TKIs are under way.

Assuming that lung cancer is responsive to hormonal manipulations, another modality to alter estrogen signaling could be via estrogen synthesis inhibition. Aromatase is a cytochrome P450 enzyme complex that mediates the final, rate-limiting step in estrogen synthesis. More specifically, aromatase catalyzes three consecutive hydroxylation reactions converting C19 androgens to aromatic C18 estrogens. Upon receiving electrons from NADPH-cytochrome P450 reductase, aromatase converts androstenedione and testosterone to estrone and estradiol, respectively [19]. Apart from its expression in the ovaries, the enzyme is also present in other tissues such as breast, lung, liver and brain. Aromatase expression has been recently demonstrated in NSCLC tumour specimens [5,7], which could represent a local source of estrogen production in lung cancer tissue in both genders.

Aromatase inhibitors (AIs) might also be effective in NSCLC by reducing the biosynthesis of estrogens and thereby inhibiting estrogen-depended pathways in lung tumours. Currently, there are two classes of third-generation aromatase inhibitors: reversible nonsteroidal inhibitors, such as anastrozole and letrozole, and irreversible steroidal inactivators, such as exemestane. Exemestane inhibits aromatization *in vivo *by about 98 percent in postmenopausal breast cancer patients. In the adjuvant setting, switching to exemestane after 2-3 years of adjuvant tamoxifen was more effective than continuing tamoxifen [20]. In this trial, patients assigned to exemestane displayed a trend of lower incidence of subsequent primary lung cancer compared to those maintained on tamoxifen (4 vs 12).

In the current study, we investigated the anti-tumour activity of exemestane in lung cancer cell lines H23 and A549 on cell proliferation and apoptosis. In addition, we examined the effect of exemestane on aromatase activity and expression levels. We also tested the hypothesis that exemestane might exert its effect on aromatase activity through a cAMP-dependent mechanism. Finally, we evaluated the effect of exemestane on EGFR localization and activation.

## Results

*In vitro *experiments were performed in two NSCLC cell lines, H23 and A549. Both cell lines express ERα and ERβ [3,5,7]. H23 cells express higher levels of ERα compared to A549 cells, whereas the latter cell line expresses higher levels of ERβ compared to H23 cells [7].

### Aromatase expression in H23 and A549 cells

**Figure 1:**
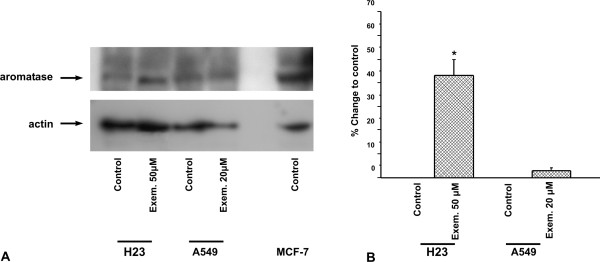
**The effect of exemestane on protein levels of aromatase in H23 and A549 cells**. **A**: H23 and A549 cells were treated with 50 μM and 20 μM exemestane respectively and 48 h later, cells were lysed and analyzed in SDS-PAGE. Actin was used as an internal control. The figure is a representative from at least three independent experiments. **B**: Quantification of western blot images. The results are expressed as % change of control. Asterisks denote a statistically significant difference (unpaired *t*-test) compared to control. * *P *< 0.05.

**Figure 2:**
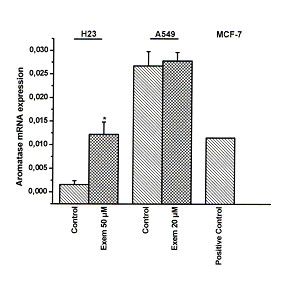
**The effect of exemestane on mRNA levels of aromatase in H23 and A549 cells**. H23 and A549 cells were treated with 50 μM and 20 μM exemestane respectively and 48 h later, aromatase mRNA levels were studied with real time RT-PCR. Results are expressed as relative expression and normalized to untreated cells. Asterisks denote a statistically significant difference (unpaired *t*-test) compared to untreated cells. * *P *< 0.05.

A549 cells express higher protein and mRNA aromatase levels than H23 cells (Fig. 1 and Fig. 2). Aromatase activity was detected in both cell lines. Exemestane did not affect protein nor mRNA levels of aromatase in A549 cells, 48 h after its application. In contrast, protein and mRNA levels were increased in H23 cells, 48 h after its application (Fig. 1 and Fig. 2).

### Exemestane inhibited H23 and A549 cell growth and increased apoptosis

**Figure 3:**
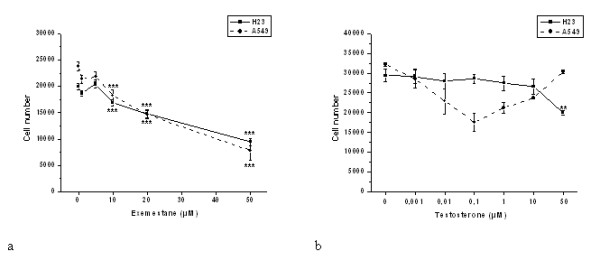
**Dose response of a) exemestane and b) testosterone in H23 and A549 cells**. Different doses of exemestane or testosterone were applied on H23 and A549 cells and 48 h later, the number of cells was estimated with the colorimetric MTT assay. Results are expressed as mean ± SEM of the number of cells from at least three independent experiments performed in triplicates. Asterisks denote a statistically significant difference (unpaired *t*-test) compared to untreated cells. **P < 0.01 and ***P < 0.001.

We studied the effect of exemestane on the proliferation of the human NSCLC cell lines, H23 and A549. Exemestane inhibited cell proliferation in both cell lines in a dose dependent manner, 48 h after its application (Fig. 3a). Fifty percent growth inhibition was recorded at 50 μM for H23 and 20 μM for A549. All further experiments for each cell line were performed with these concentrations of exemestane. Moreover, evaluating the effect of testosterone on proliferation of H23 and A549 cells, we found that testosterone did not affect cell proliferation at lower doses whereas it decreased cell number at higher doses (Fig. 3b).

**Figure 4:**
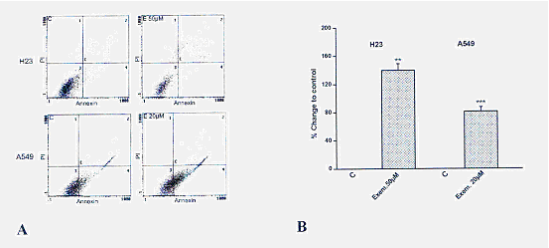
**The effect of exemestane on apoptosis of H23 and A549 cells**. A: Flow cytometry analysis of H23 and A549 following exposure to exemestane. A representative image from three independent experiments is shown. H23 cells. C: Control and E 50 μM: Exemestane 50 μM. A549 cells. C: Control and E 20 μM: Exemestane 20 μM. B: Results are expressed as the % percentage of Annexin^+ ^cells ± SEM compared to untreated cells from at least three independent experiments. Asterisks denote a statistically significant difference (unpaired *t*-test) compared to untreated cells (C). ** *P *< 0.01 and *** *P *< 0.001.

Furthermore, the percentage of annexin^+ ^cells increased 48 h after treatment of H23 or A549 with exemestane compared to untreated cells (Fig. 4). Although there are data regarding cell cycle arrest by aromatase inhibitors in breast cancer cells [21], a similar effect was not found with exemestane in NSCLC cell lines in our study (data not shown).

### Aromatase activity in H23 and A549 cells

**Figure 5:**
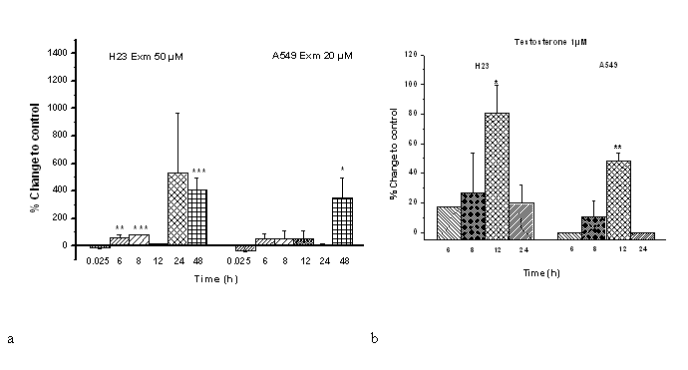
**The effect of a) exemestane and b) testosterone on aromatase activity of H23 and A549 cells**. H23 and A549 cells were treated with 50 μM and 20 μM exemestane, respectively and both cell lines were treated with 1 μM testosterone. At the time points of 15 min, 6, 8, 12, 24 and 48 h, samples were analysed as described in Methods. *P < 0.05, ** *P *< 0.01 and *** *P *< 0.001.

As aromatase catalyses the conversion of androstenedione and testosterone to estrone and estradiol, respectively, its activity was determined by the production of estradiol after treatment of cells with exemestane. H23 and A549 cells were treated with exemestane without exogenous addition of testosterone at the indicated concentrations, and 15 min later estradiol production was measured. Previous reports have indicated that the estimated time for achieving 50% of aromatase inhibition by exemestane is 13.9 min [19,22]. We found that a 15 min treatment with exemestane caused a non-significant reduction of estradiol levels in both cell lines (Fig. 5a). However, estradiol levels were increased 6, 8, 12, 24 and 48 h later in both cell lines (Fig. 5a).

Furthermore, aromatase activity was determined at 6, 8, 12 and 24 h, following treatment of both cell lines with 1 μM testosterone. This experiment was used as an internal control. The time point of 15 min was not used since there are previous data showing that in breast cancer cells, aromatase is activated 6 h after incubation with a suitable substrate [23]. As was expected, we found that application of testosterone increased the levels of estradiol (Fig. 5b), with the maximum production of estradiol being observed 12 h after treatment of cells with the substrate.

### cAMP levels in H23 and A549 cells

**Figure 6:**
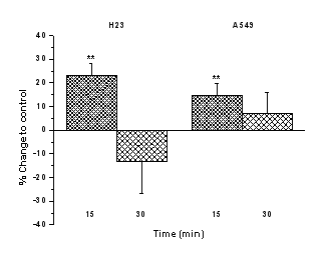
**The effect of exemestane on cAMP levels in H23 and A549 cells**. H23 and A549 cells were treated with 50 μM and 20 μM exemestane, respectively. At the time points of 15 and 30 min, samples were analysed as described in Methods. ** *P *< 0.01.

Evaluating the effect of exemestane on cAMP levels, we found an increase of cAMP levels in both cell lines, 15 min after treatment of cells with exemestane. This effect was reversed 30 min after the application of exemestane (Fig. 6).

### Localization and phosphorylation levels of EGFR in H23 and A549 cells

**Figure 7:**
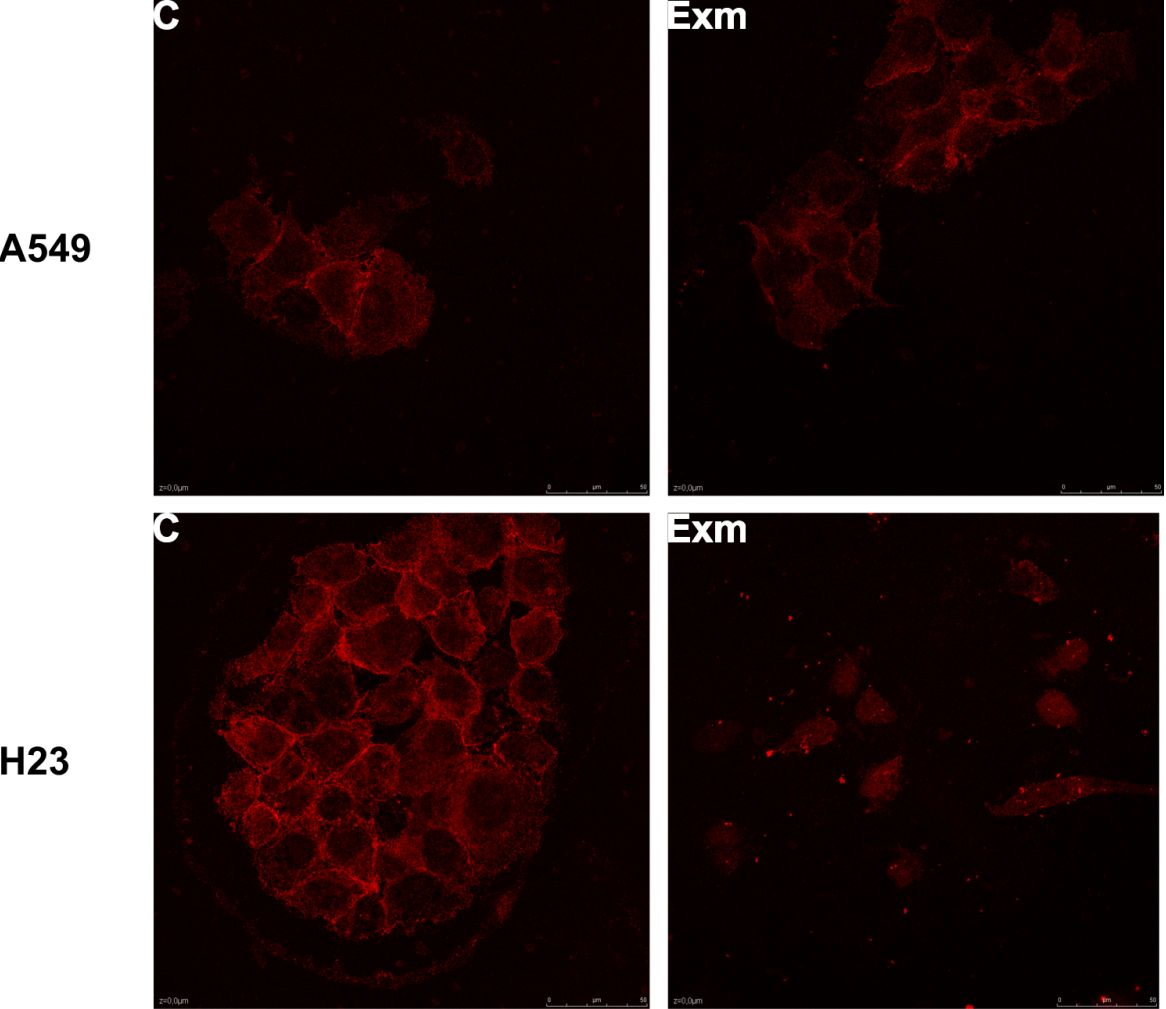
**The effect of exemestane on EGFR localization in H23 and A549 cells**. Both cell lines were treated with 50 μM and 20 μM exemestane, respectively. At the time point of 24 h, samples were analyzed as described in Methods. The figure is a representative from at least three independent experiments (magnification at 60×).

**Figure 8:**
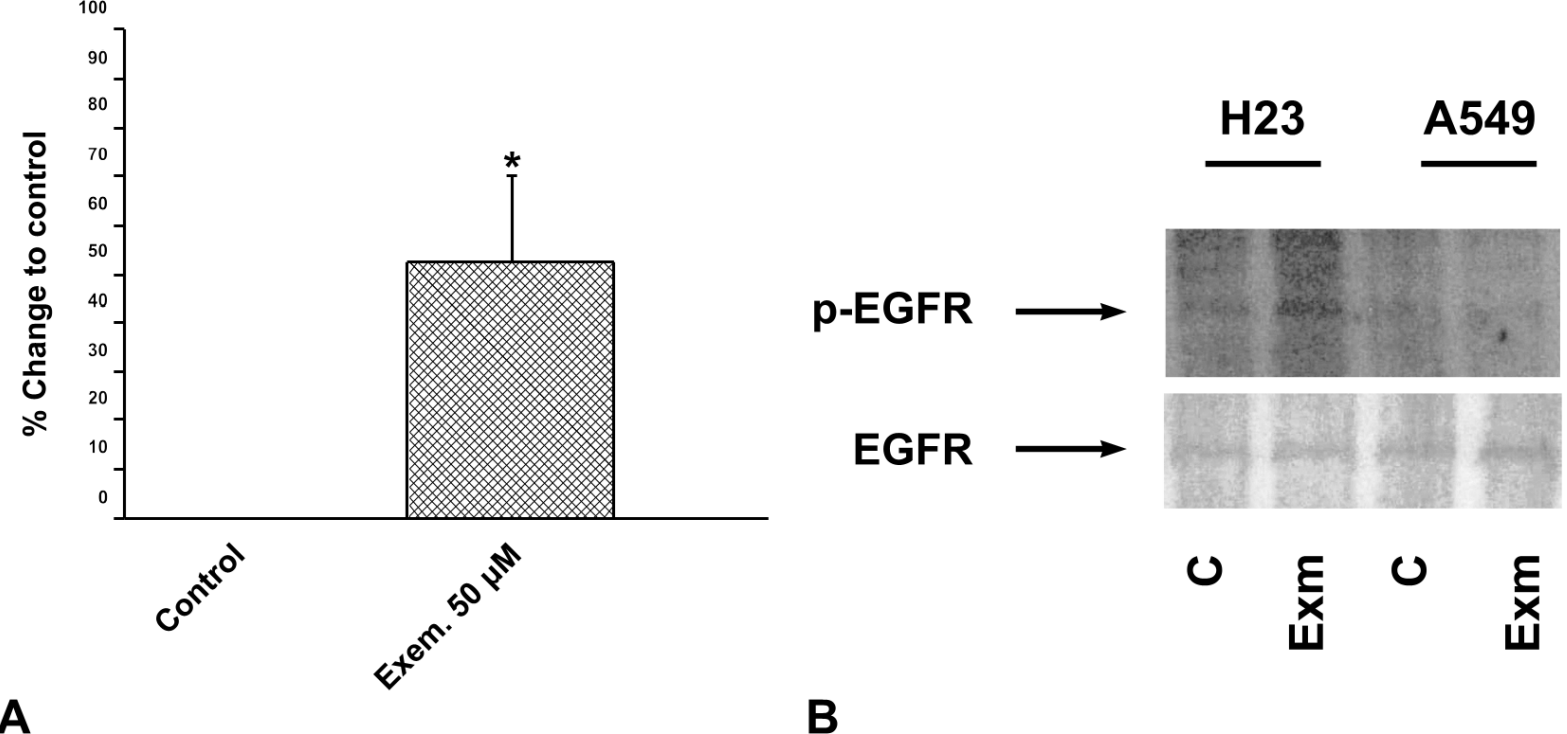
**The effect of exemestane on EGFR phosphorylation a) in H23 cells using an ELISA kit assay and b) in H23 and A549 cells using immunoblotting**. H23 and A549 cells were treated with 50 μM and 20 μM exemestane, respectively and 15 min later they were analyzed as described in Methods. C: untreated cells (H23 or A549 cells) and Exm: H23 or A549 cells treated with 50 μM or 20 μM exemestane, respectively. *P < 0.05.

Finally, we evaluated the effect of exemestane on EGFR localization and activation. In A549 cells, no changes in EGFR localization (Fig. 7) or phosphorylation (data not shown) were demonstrated following treatment of cells with exemestane. However, in H23 cells we found a translocation of EGFR from the cell membrane, 24 h after the addition of exemestane (Fig. 7). Moreover, we demonstrated an increase in EGFR activation, 15 min after treatment of H23 cells with exemestane (Fig. 8a and 8b). EGFR activation was detected using an Elisa kit assay (Fig. 8a), and the results were confirmed by immunoblot analysis (Fig. 8b).

## Discussion

In the current study, we evaluated the activity of exemestane in NSCLC cell lines H23 and A549. Our results are in agreement with previous studies [5,7] which have reported that aromatase is expressed by NSCLC cells. Lung cancer cells may use estrogens produced via aromatase as a way to maintain or even increase the ER signaling [24]. We have also found that exemestane inhibited cell proliferation in both cell lines in a dose dependent manner and apoptosis was increased without affecting cell cycle. Previous reports have indicated that AIs such as anastrozole and letrozole may inhibit proliferation of the breast cancer cell line MCF-7 through induction of apoptosis and cell cycle arrest [21].

In order to determine the effective dose of exemestane regarding cell proliferation, a set of dose dependent experiments were performed. The dose dependent curves revealed that 50% growth inhibition was recorded at 50 μM for H23 and 20 μM for A549 cells. Although the selected concentrations seem to be high, similar concentrations of an AI have also been used in previous studies for in vitro experiments [7,25]. The dose of exemestane currently used in clinical practice is 25 mg daily. Exemestane exhibits an excellent safety profile in humans, having no significant toxicity at doses up to 600 mg/day and it is exceptionally well tolerated. The maximum tolerated dose and dose limiting toxicities have yet to be identified [26]. At a single dose of 25 mg, the average peak plasma level is 18 ng/ml (approximately 0.06 nM), within 2 h post dosing. This concentration is clearly lower compared to our in vitro experimental model, as well as to the majority of other in vitro experimental models. However, the aim of our study was to focus on the effect of exemestane on aromatase activity and EGFR signalling, when used at a dose with a significant effect on lung cancer cell proliferation.

In another study which also investigated the efficacy of an AI in NSCLC, aromatase activity was inhibited by anastrozole in NSCLC cell lines, and treatment of tumour cells with anastrozole led to suppression of cell growth [7]. It is of interest that the use of an aromatase inhibitor has the same effect on cell growth despite the different nature of the inhibitors used. In fact, anastrozole is a reversible nonsteroidal inhibitor whereas exemestane is an irreversible steroidal inactivator. In addition, our data provide information with respect to the effect of exemestane on protein and mRNA levels of aromatase in NSCLC cell lines.

Previous data regarding the effect of estradiol on the proliferation of H23 and A549 cells are contradictory. When estradiol was used at concentrations up to 1 μM, no effect on cell proliferation was demonstrated [27]. In contrast, estradiol enhanced cell proliferation when working concentrations were up to 20 μM [7]. Therefore, we decided to treat both cell lines with various concentrations of testosterone, an aromatase substrate. We found that administration of testosterone did not affect the proliferation of cells. However, treatment with testosterone at higher doses was associated with a decrease in cell proliferation. In view of the fact that testosterone is converted to estradiol by aromatase, the latter finding seems conflicting. However, previous reports have indicated that estrogens may reduce cell proliferation. Recent data suggest that estrogens are capable of inducing apoptosis in certain cell types, including breast cancer cells, immune system cells and bone-derived cells [28,29].

Although exemestane showed a trend to inhibit aromatase activity 15 min after its application in both cell cultures, this inhibitory effect was reversed 6 h later. Previous reports with aminoglutethimide, an AI, also showed an increase in aromatase activity in breast cancer cell line SK-BR-3, choriocarcinoma cell line JAR, and in hepatocellular carcinoma HepG2 [30]. At least three mechanisms have been suggested. The first is that AIs can stabilize aromatase protein by forming enzyme-inhibitor complexes that slow down the degradation of the enzyme [31]. Indeed, we found an increase of aromatase protein levels in H23 cells. Another mechanism is that AIs can modulate aromatase expression at the transcription level [30]. In our study, we observed an increase of aromatase mRNA levels in H23 cells. On the other hand, we didn't find the same changes in protein and mRNA levels in A549 cells. The third possible mechanism includes changes in aromatase activity through a cAMP-dependent mechanism, without affecting mRNA or protein expression of the enzyme [30]. Indeed, in our study we demonstrated that treatment of cells with exemestane increased the levels of cAMP in both cell lines, 15 min after the application of the agent. These findings suggest that exemestane may increase the activity of aromatase through a cAMP-dependent mechanism.

Although the decrease in aromatase activity was assessed 15 min following exemestane application, and this effect was reversed 6 h after the administration of the drug, changes in cell proliferation and aromatase protein and mRNA levels were measured 48 h after the addition of exemestane. Based on the information that the half life of aromatase protein is 28.2 h [32], we believe that the results from the inhibitory effect of exemestane could be observed 48 h after drug application.

In a recently reported study [24] that assessed the prognostic role of aromatase expression in NSCLC patients, lower levels of the enzyme predicted a higher probability of survival in women aged 65 years or older. These findings suggest that older female patients with NSCLC and high levels of aromatase might represent a group of patients where treatment with an AI could be beneficial. In our study, we showed that the cell line A549, which expresses higher aromatase protein and mRNA levels might be a better target for exemestane, since aromatase expression was not increased by this agent, in contrast to the cell line H23 with lower aromatase levels. Moreover, fifty percent growth inhibition was recorded at 50 μM for H23 and 20 μM for A549 cells.

Finally, we found that the administration of exemestane in H23 cells was associated with EGFR activation, 15 min after drug application. Current evidence suggests that cross-talk between steroid receptors and growth factor receptors exists in a bidirectional way. In addition, significant interactions between estrogen signaling and EGFR have been recognized not only in breast, but also in NSCLC [5]. Exemestane may affect EGFR pathway independently of aromatase inhibition. It has been demonstrated that exemestane can activate ER [33] which in turn leads to the production of the EGFR ligand amphiregulin, resulting in EGFR activation [34]. This mechanism may be implicated in exemestane resistance and may also be involved in exemestane-induced EGFR activation found in our study. Other interactions between growth factor signaling pathways and aromatase in NSCLC have also been reported [35]. The effect of exemestane on EGFR activation observed in our study also suggests that this agent may be less effective in H23 cells, compared to A549 cells. In addition, the combination of exemestane with an anti-EGFR agent may represent a more efficient option in H23 cells. Recently, we investigated the dual inhibition of aromatase and EGFR in NSCLC cell lines using exemestane and the EGFR tyrosine kinase inhibitor erlotinib. In agreement with the aforementioned results, the combination of exemestane and erlotinib was more effective than each agent alone, in H23 cells [36].

The activation of EGFR followed by its translocation may lead to receptor internalization and proteolytic degradation [37]. Alternatively, EGFR has been detected in caveolae, Golgi complex, endoplasmatic reticulum, lysosome-like structures, nuclear envelope, nucleus and mitochondria [38,39]. In addition, it has been demonstrated that EGFR mitochondrial localization is implicated in cell survival [39]. In agreement with this observation, unpublished data from our laboratory confirmed the translocation of EGFR to mitochondria in H23 cells, following treatment with exemestane. Moreover, this translocation was inhibited by the concurrent administration of erlotinib.

In summary, our results suggest that exemestane might elevate estradiol levels in both cell lines through a cAMP-dependent mechanism. In addition, exemestane enhances estradiol levels in H23 cells through an increase in mRNA and protein levels of aromatase. However, the antiproliferative effect of exemestane through induction of apoptosis remains to be elucidated. A recent report demonstrated that high concentrations of estradiol, under low growth stimulated conditions, decrease cell proliferation and increase apoptosis in breast cancer cells through the sustained activation of JNK pathway [40]. Indeed, our experiments were performed under low growth stimulated conditions which mimic the low estrogenic milieu in postmenopausal women [40]. This mechanism may represent a possible explanation for the antiproliferative effect of exemestane in both NSCLC cell lines, found in our study.

## Conclusion

In conclusion, our study provides evidence that aromatase is expressed by NSCLC cells and exemestane inhibits cell proliferation. Moreover, levels of aromatase expression might have a predictive ability in the activity of this agent, since exemestane may activate EGFR pathway in cells with low levels of aromatase. Further studies are needed to investigate the effectiveness of exemestane in NSCLC and evaluate the potential for incorporating AIs in the management of lung cancer patients.

## Methods

### Cell culture and reagents

NSCLC cell lines H23 and A549, and control breast cancer cell line MCF-7 were purchased from American Type Culture Collection (ATCC). H23 and A549 cells were cultured in RPMI 1640 medium with 2 mM L-glutamine and supplemented with 1 mM sodium pyruvate, 4.5 g/L glucose, 1.5 g/L sodium bicarbonate and 10% fetal bovine serum. MCF-7 cells were cultured in Eagle's Minimum Essential medium (EMEM) with Earle's BSS and 2 mM L-glutamine and supplemented with 1.0 mM sodium pyruvate, 0.1 mM nonessential amino acids, 1.5 g/L sodium bicarbonate, 0.01 mg/ml bovine insulin and 10% fetal bovine serum. Cells were cultured at 37°C, 5% CO_2 _and 100% humidity.

The aromatase inhibitor exemestane (Aromasin) was kindly provided by Pfizer. Exemestane was applied to cell lines after cell attachment at doses of 1, 5, 10, 20 and 50 μM. Testosterone was purchased by Sigma (Sigma, Steinheim, Germany) and was applied to cell lines after cell attachment at doses of 0.001, 0.01, 0.1, 1, 10 and 50 μM. Exemestane was diluted in dimethyl sulfoxide (DMSO) and testosterone was diluted in ethanol. The final concentration of both DMSO and ethanol in culture medium was 0.5%. After reaching 50% confluence, cells were washed with phosphate buffer saline (PBS) and incubated with phenol red-free medium with 1% dextran-coated, charcoal-treated FBS (working medium) for 24 h to deplete estrogen [7]. All the experiments were performed according to these conditions. Thereafter, cells were treated with exemestane or testosterone at the indicated time points and doses.

### Immunoblotting

Cells were plated at Petri dishes. After reaching 80% confluence, cells were treated with working medium as described above. Forty eight hours after exemestane addition, cells were collected with scrapper and lysed using appropriate lysis buffer (50 mM Tris-HCl pH 7.5, 150 mM NaCl, 5 mM EDTA, 1% Triton, 10% glycerol, 1 mM phenylmethyl-sulphonyl-fluoride, 2 mM Na-orthovanadate and 10 mM leupeptin). Protein concentration was determined by Bradford assay. Samples were analyzed by immunoblotting as described previously [7]. Actin was used as control. A goat polyclonal anti-aromatase antibody (CYP19) C16 (dilution 1:5000, Santa Cruz, CA, USA) and a monoclonal anti-actin antibody were used (dilution 1:1000, Chemicon, Millipore, Temecula, CA, USA). For the detection of phosphorylated EGFR/EGFR, an immunoprecipitation was performed prior to immunoblotting. Briefly, cells were prepared as described above. Fifteen minutes after the addition of exemestane, cells were collected with scrapper and lysed with sonication (3 cycles for 5 sec each, MSE PG 616) using the same lysis buffer as above. Protein concentration was determined and 1 mg total protein was immunoprecipitated with a monoclonal anti- EGFR antibody (dilution 1 μg per 0.5 mg of total protein, 3H2094, Santacruz, CA, USA), overnight at 4°C under continuous agitation. In each sample, 50 μl of protein-A sepharose beads (Calbiochem, Merck, Dermstadt, Germany) were added and samples were incubated for 4 h, at 4°C under continuous agitation. Precipitates were washed twice with ice-cold lysis buffer and sepharose beads were resuspended in 50 μl 2× sample buffer (0.5 M Tris-HCl pH 6.8, 20% glycerol, 2% SDS and 2% bromophenol blue, 10% b-mercaptoethanol). Samples were heated for 5 min at 95°C and analyzed by immunoblotting. A monoclonal anti-tyrosine antibody (dilution 2 μg/ml, Upstate, Lake Placid, NY, USA) and a monoclonal anti-EGFR antibody (dilution 1:200, 3H2094, Santacruz, CA, USA) were used. Detection of immunoreactive proteins was performed by chemiluminescence using horseradish peroxidase substrate SuperSignal (Pierce, Rockford, IIL, USA), according to manufacturer's instructions.

### RNA isolation and cDNA synthesis

Total RNA was extracted from cells using Absolutely RNA RT-PCR kit (Stratagene, La Jolla, USA) according to the manufacturer's instructions. Integrity of RNA was confirmed by visualization of ribosomal bands by EtBr-stained agarose gel electrophoresis. RNA was quantified using Ribogreen (Molecular Probes, Leiden, the Netherlands) and the MX3000p (Stratagene, La Jolla, USA) according to manufacturer's instructions. First strand cDNA was synthesized as previously described [41].

### Real time PCR

Quantification of cyp19 mRNA was performed using gene-specific primers (F: 5'-AACAACTCGACCCTTCTTTATG-3', R: 5'-TTTGAGGGATTCAGCACAG-3') and SYBR Green I intercalation dye in Brilliant Sybr Green QPCR Master Mix (Stratagene, La Jolla, USA). Expressed Alu-Sq repeat levels were also similarly quantified using primers designed by Dr J. Vandesompele, Gent University Hospital, Belgium. In addition, a standard curve was included in each run for assay validation. Reactions were performed as previously described [41]. Cyp19 mRNA levels were normalised to Alu-Sq levels which were found to be unaffected by exemestane treatment.

### Cell proliferation assay

To determine whether exemestane or testosterone affect the proliferation of H23 and A549 NSCLC cell lines, the 3- [4,5-dimethylthiazol-2-yl]-2,5-dimethyltetrazolium bromide (MTT) assay was used, as previously described [42]. Briefly, cells were plated at a density of 2 × 10^4 ^cells per well in 24-well tissue culture plates. The medium was aspirated later at 24 h, cells were washed twice with phosphate buffer saline (PBS) and 0.5 ml of working medium was added in each well. After a 24 h incubation exemestane or testosterone was added into cells. Cell proliferation was measured 48 h after exemestane addition using MTT assay. MTT stock (5 mg/ml in PBS) at a volume equal to 1/10 of the medium was added and plates were incubated at 37°C for 2 h. The medium was removed, the cells were washed with PBS pH 7.4 and 100 μl acidified isopropanol (0.33 ml HCl in 100 ml isopropanol) was added to each well and agitated thoroughly in order to solubilise the dark blue formazan crystals. The solution was transferred to 96-well plates and immediately read on a microplate reader (Tecan, Sunrise, Magellan 2) at a wavelength of 570 nm. Results were always confirmed by direct measurements of the cells using a standard Neubauer haemocytometer.

### Apoptosis assay

Both NSCLC cell lines were plated at 1 × 10^5 ^cells per well in 6-well plates. Exemestane was added as previously described. At the end of a 48 h incubation, cells were washed twice with PBS, trypsinized for 6 min and centrifuged for 4 min at 166 g. Cells were resuspended in 200 μl 1× binding buffer (10 mM HEPES pH 7.4, 140 mM NaCl, 2.5 mM CaCl_2_). The cell suspension was incubated with 5 μl Annexin V-FITC in the dark at 25°C, for 10 min. Then, 10 μl of the 20 μg/ml propidium iodide stock solution was added, followed by 400 μl of binding buffer and cells were immediately analyzed by flow cytometry [43] (EPICS-XL of Coulter) according to manufacturer's instructions (rh Annexin V/FITC kit, Bender MedSystems).

### Aromatase activity

Aromatase activity was estimated through measurement of estradiol production by ELISA (IBL Hamburg, Germany) according to manufacturer's instructions at 450 nm with a microplate reader (Tecan, Sunrise, Magellan 2). Briefly, cells were plated at 2 × 10^4 ^cells per well in 24-well plates. Exemestane or testosterone was added as previously described. At several time points after incubation (0.25, 6, 8, 12, 24 and 48 h), supernatants were collected and stored at -20°C until the end of the experiment. Twenty five μl of each sample was added into well that was pre-coated with a polyclonal antibody against estradiol molecule. Then 200 μl of appropriate enzyme conjugate was added to each well and an incubation of 2 h was followed. After the incubation, wells were washed 3 times with wash solution and 100 μl of suitable substrate solution was added to each well. Fifteen minutes later, the enzymatic reaction was stopped by adding 50 μl of stop solution. The absorbance of each well was measured at 450 nm with a microplate reader within 10 min after adding the stop solution. An appropriate standard curve was determined.

### cAMP detection

The cAMP levels were measured using an ELISA kit assay (R&D Systems Europe, Ltd, UK). Samples were prepared according to manufacturer's instructions. Briefly, H23 and A549 cells were plated in Petri dishes. After reaching 50% confluence, cells were treated with working medium as described above and 24 h later exemestane was added into cells. Supernatants were removed 15 and 30 min later and cells were washed 3 times with cold PBS. Then, cells were resuspended in cell lysis buffer 5 (1×) that was included in kit to a concentration of 1 × 10^7 ^cells/ml. Cells were freezed at ≤ -20°C and thawed with gentle mixing. The cycle of freeze/thaw was repeated once. Samples were centrifuged at 600 × g for 10 min at 4°C to remove cellular debris. Samples were stored at -20°C until their use. Suitable microplate containg pre-coated wells with goat anti-mouse antibody, was incubated with 50 μl of a monoclonal antibody specific for cAMP for 1 h at room temperature. After the incubation, wells were washed 3 times with wash solution and 100 μl of each sample was added to each well. Then, 50 μl of cAMP congjugate was added to all wells and an incubation of 2 h was followed. After a step of 3 washes, 200 μl of a substrate solution was added to each well and an incubation of 30 min in dark was followed. Then, 100 μl of stop solution was added to each well. The absorbance of each well was measured at 450 nm with correction at 570 nm in a microplate reader (Tecan, Sunrise, Magellan 2) within 30 min after adding the stop solution. An appropriate standard curve was determined.

### Immunofluorescense

Both NSCLC cell lines were treated with exemestane as previously described. Twenty four hours later, medium was removed and cells were washed twice with PBS. Cells were fixed with a 4% paraformaldehyde in PBS buffered solution for 10 min at room temperature and then they were rinsed 3 × 5 min with PBS. An incubation of 1 h was followed by a 3% BSA solution supplemented with 10% FBS at 37°C. After the incubation with blocking solution, cells were rinsed once with PBS for 5 min and they were treated overnight at 4°C with a monoclonal anti-EGFR antibody diluted in blocking solution (10 μg/ml, Upstate, Lake Placid, NY, USA,). Cells were rinsed 3 × 5 min with PBS and then an anti-mouse antibody conjugated with Alexa Fluor 594 (1:500, Invitrogen, Molecular probe) diluted in blocking solution was added for 30 min at 37°C. Cells were rinsed 3 × 5 min with PBS and mounted on glass sides. Fluorescence was visualized using a Leica microscope.

### EGFR phosphorylation

The levels of phosphorylated EGFR were determined using an ELISA kit system (Bender MedSystems GmbH, Austria), according to the manufacturer's instructions. Briefly, cells were seeded in 100 mm petri dishes at a density of 10^6 ^cells per dish and were treated as previously described. Fifteen minutes after the addition of exemestane, supernatants were removed and cells were collected with scraper. Lysis of cells was followed by adding receptor binding buffer. Samples were transferred onto 96- microwell plate coated with a monoclonal antibody to human active EGFR and incubated for 1 h at 37°C. Microwells were emptied and washed three times with washing buffer. An anti-phosphotyrosine monoclonal antibody (horseradish peroxidase conjugated) was added and samples were incubated for 1 h at 37°C. Microwells were washed four times and a suitable substrate solution was added to each sample for 15 min. The reaction was stopped by adding a stop solution and the samples were immediately measured on a microplate reader (Tecan, Sunrise, Magellan 2) at a wavelength of 450 nm. The results were normalized by measuring the amount of total proteins using Bradford assay, since total protein amount is not altered for the time point of 15 min after exemestane addition.

### Statistical analysis

Differences between groups and controls were tested by unpaired *t*-test. Each experiment included at least triplicate measurements. All results are expressed as mean ± SEM from at least three independent experiments.

## Competing interests

The authors declare that they have no competing interests.

## Authors' contributions

AKK participated in the conception, design and coordination of the study, interpretation of data, and drafted the manuscript. EG participated in the design of the study and interpretation of data, performed the analyses, and was involved in drafting the manuscript. KI and AA contributed to the analyses and interpretation of data. TRJE and AGP critically revised the manuscript. HPK participated in the design and coordination of the study. All authors have read and approved the final version of the manuscript.
